# Primary Endoscopic Stapes Surgery: A Comparison of Adipose Tissue and Gelfoam Seal

**DOI:** 10.1007/s12070-020-02207-y

**Published:** 2020-10-23

**Authors:** Pradeep Pradhan, Anindya Nayak, Sidharth Pradhan, Prity Sharma, Chappity Preetam, Pradipta Kumar Parida

**Affiliations:** Department of ENT and Head Neck Surgery, All India Institute of Medical Sciences (AIIMS), Bhubaneswar, Odisha 751019 India

**Keywords:** Endoscopic stapes surgery, Adipose tissue seal, Gelfoam seal, Comparison

## Abstract

To compare the efficacy between the commonly used sealing materials, i.e., adipose tissue and the gelfoam in primary endoscopic stapedotomy. Lobular fat and gelfoam have been used in patients who underwent endoscopic stapedotomy between two groups, each containing 29 patients. The hearing outcomes and postoperative complications were compared at the end of 12 weeks between two groups. The ABG of ≤ 10 dB was achieved in 69% of cases in group A and 76% of cases in group B. There was a significant short-term (1 week) improvement in the Dizziness Handicap Inventory score (*p* = 00) with patients of adipose tissue seal compared to the gelfoam. Although the audiological outcomes were comparable between the two groups, the use of the adipose tissue can be a better alternative than gelfoam to control vertigo in the early postoperative period without causing any significant morbidity to the patient.

## Introduction

Because of the enhanced visualization of the middle ear, endoscopic stapedotomy has become more popular in recent times. It ensures the preservation of the posterior canal wall along with the chorda tympani nerve in the majority of cases [1–3]. The improved visual field could have been possible due to the advancement of the rigid endoscopes for the inspection of the detailed middle ear structures far away from the surgical field [4, 5]. Although satisfactory clinical and audiological results have been obtained in endoscopic stapes surgery as reported in the literature [6], the vestibular dysfunction (giddiness) in the postoperative period is rarely described. Various autologous and synthetic materials have been used in the past to seal the oval window to overcome the commonest complication, i.e., giddiness resulted in inconsistent control over vertigo in spite of similar hearing outcomes [7, 8]. Amongst all, gelfoam and fat are common sealing materials used for the closure of the stapes fenestra irrespective of the surgical techniques. Autologous fat, because of its better sealing properties, could be more effective in controlling postoperative vertigo besides significant improvement in hearing [8]. In the present study, we have compared the efficacy between the commonly used sealing materials, i.e., adipose tissue and the gelfoam, with concern to the audiological and vestibular outcome in the postoperative period in patients undergoing primary endoscopic stapes surgery.

## Materials and Methods

**Fig. 1:**
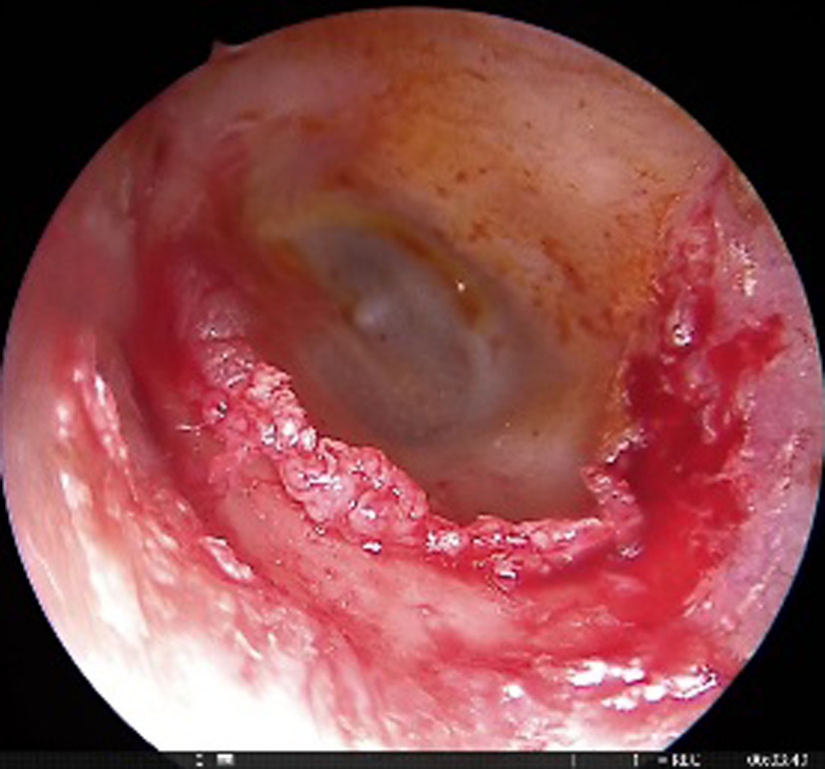
Transcanal incision given (Right ear) from 6 o’clock to 12 o’clock poison

**Fig. 2:**
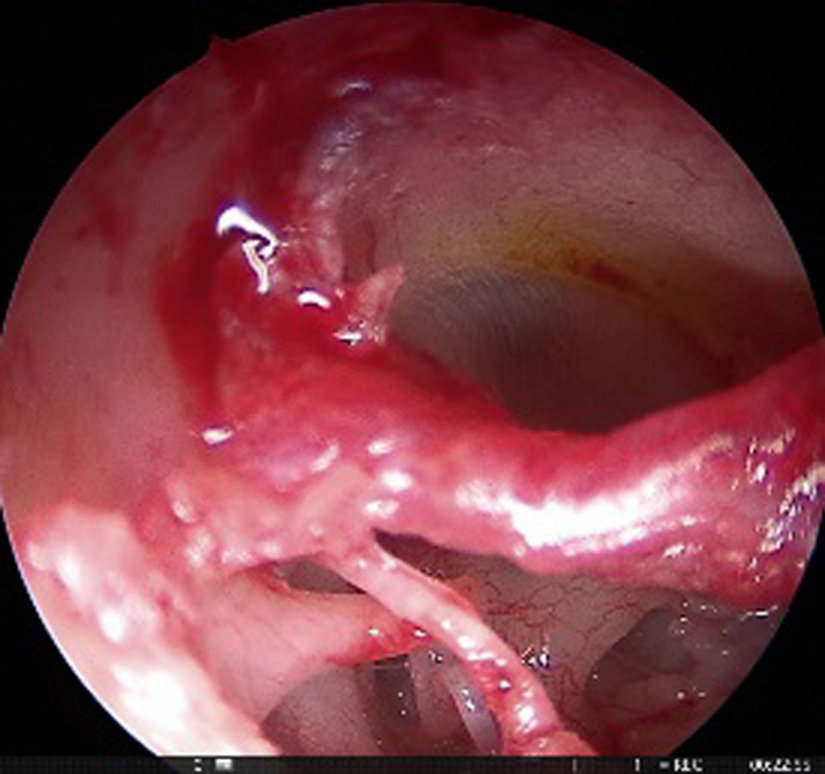
Complete exposure of stapes suprastructure

**Fig. 3:**
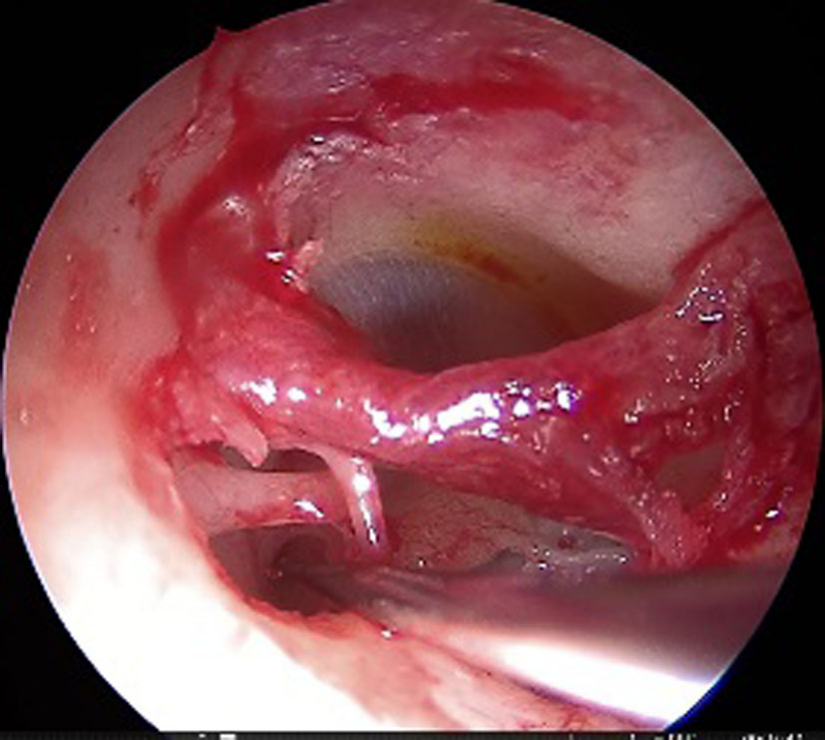
Fenestra was made over the stapes footplate with 0.8 mm perforator

**Fig. 4:**
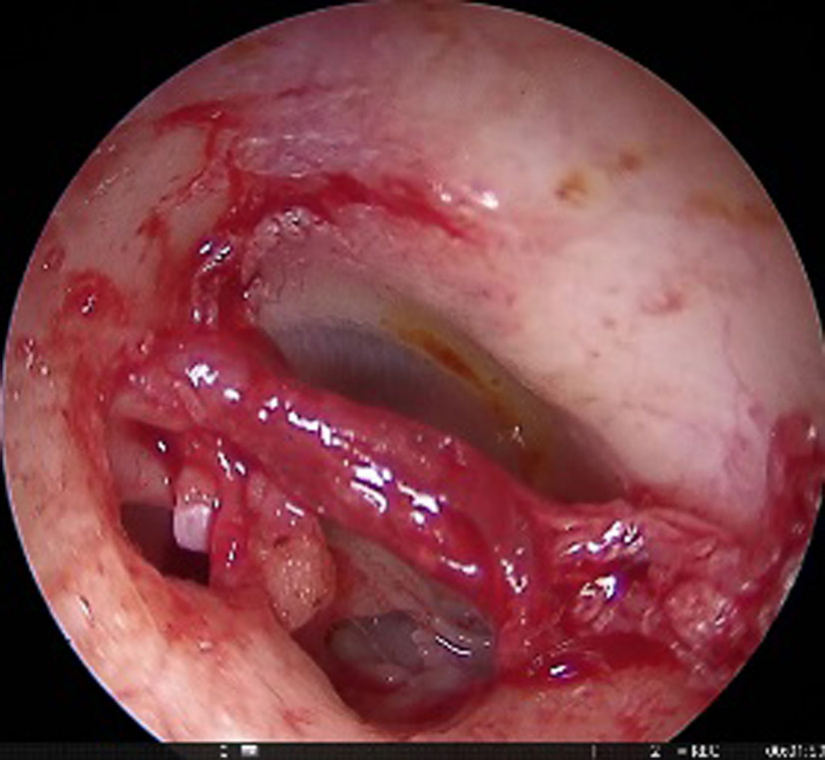
Teflon piston (0.6 mm diameter, 4.25 mm length) was inserted through the stapedial window, anchored over the long process of incus, and a piece of lobular fat was used to seal the stapedial window after the insertion of the piston

This was a retrospective study of patients who were operated in the Department of Otorhinolaryngology between May 2017 and Feb 2019. A total of 58 patients of bilateral otosclerosis were included in the study, which was allocated into two groups (gelfoam and fat plugging groups, each containing 29 patients each) by quasi randomization i.e., alternatively designated into either group. All the patients underwent endoscopic stapedotomy with the help of a 3 mm nasal rigid endoscope. Gelfoam and lobular fat were used for plugging of the stapes window in group A and group B, respectively, containing 29 patients each. The diagnosis was made after the detailed clinical history, otoscopic features suggesting intact tympanic membrane, and conductive deafness of more than 20 dB HL with a normal bone conduction threshold (at the frequency of 500 Hz, 1 kHz, 2 kHz, and 4 kHz) with the complete absence of the stapedial reflex. Patients presenting with mixed hearing loss were excluded from the study. The demographic and clinical profile of the patients, including the diameter of the auditory canal, pure tone audiogram (PTA), were noted in the preoperative period. Patients were counselled in the preoperative period regarding the surgical outcome and the expected postoperative complications attributed to the surgery. Written informed consent was taken for each patient before the surgery. All the patients were operated by a single surgeon under local anaesthesia. Xylocaine 2% with adrenaline was injected in the canal, covering all four quadrants. Transcanal incision was given 6 mm lateral to the annulus, from 6 o'clock to 12 o'clock position (Fig. 1). The meatal flap was elevated carefully until the middle ear cavity was reached. In selective cases, canaloplasty was performed, especially in a narrow/over hanged auditory canal. Again, the posterior canal wall was curetted in cases where there occurred incomplete visualization of the horizontal segment of the fallopian canal and the base of the pyramidal process (Fig. 2). After complete exposure of the mesotympanum, the otosclerotic focus was identified in each case. The stapedial tendon was incised with the help of a curved micro scissor. Both the anterior and posterior crus were fractured, and the suprastructure was removed with a forceps. Fenestra was made over the posterior half of the footplate with perforator (0.8 mm diameter). Teflon piston (0.6 mm diameter) was inserted in all the cases after the measurement of the vertical height from the undersurface of the incus to the footplate (Fig. 3). In 29 patients, lobular fat was used for sealing of the stapes window, and in 29 patients, gelfoam was used (Fig. 4). The intraoperative hearing was assessed in each patient after complete insertion of the prosthesis. The auditory canal was packed with medicated gelfoam. The lobular incision was stitched, and a small dressing was placed over the pinna. Each patient was assessed for the intraoperative variables like the preservation of chorda tympani nerve, removal of the bony canal, operative time, and postoperative dizziness. Six hours after surgery, each patient was assessed for the presence of vertigo with or without of nystagmus.

## Follow-Up

Patients were discharged 24 h after the surgery and advised to attend the otology clinic at 1, 4, and 12 weeks in the postoperative period. During each follow-up period, patients were subjected to otoscopic examination and PTA to assess the anatomical and audiological outcomes. At the end of 12 weeks, both the air conduction and the bone conduction thresholds were measured and later compared between the preoperative the postoperative audiogram and at the frequency of 500 Hz, 1 kHz, 2 kHz, and 4 kHz in both the groups. Similarly, the mean postoperative air–bone gap (ABG) was compared with the preoperative value. Giddiness was evaluated after 1, 4, and 12 weeks of surgery by using the dizziness handicap inventory (DHI) score and later was compared between the two groups. When the dizziness score was < 30, later was considered insignificant without affecting the routine daily activities.

### Statistical Analysis

Results were presented as n (%), mean (range), standard deviation (SD), Standard error difference (SED), Confidence interval (CI). Preoperative and postoperative data were compared using the unpaired 't' test, and Categorical variables between groups were analyzed using the Chi-squared test or Fisher's exact test. Statistical package SPSS v 20.0 was used for the statistical analysis of the data.

## Results

**Fig. 5:**
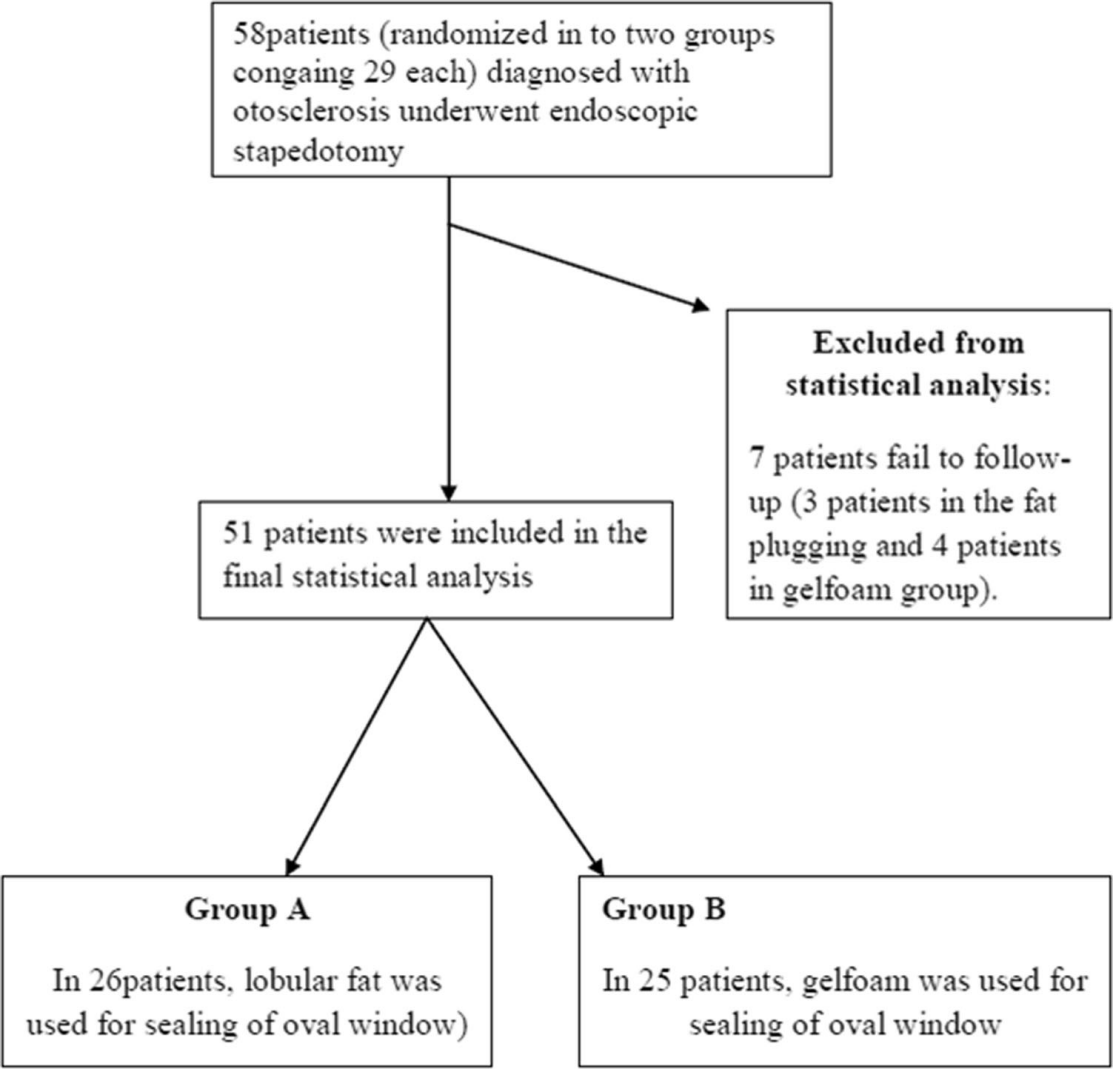
Study design

**Fig. 6:**
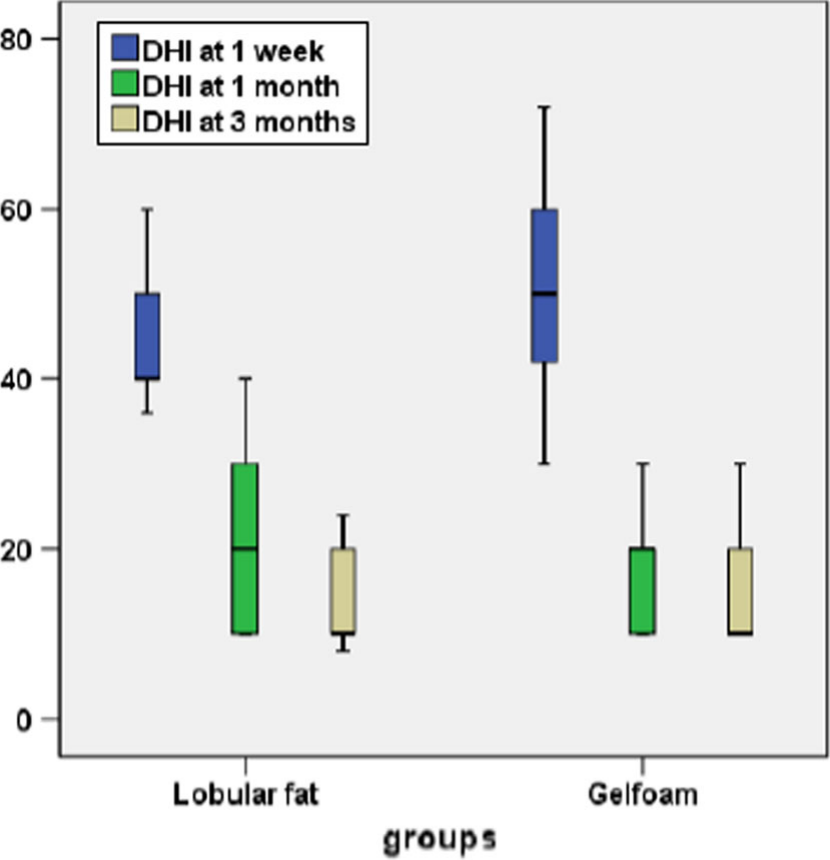
Diagram shows the comparative DHI scores between fat and gelfoam plugging at the end of 1,4 and 12 weeks in the postoperative period

A total of 58 surgeries were performed for bilateral otosclerosis using a 3 mm otoendoscope. Adipose tissue and gelfoam were used in group A and group B, respectively, for oval window sealing. During the follow-up period, 3 patients in group A and 4 patients in group B were lost; hence total 26 patients in group A and 25 patients in group B were considered for the final analysis (Fig. 5). The mean age of the patients in group A was 35 years (range 22–48), and patients in group B was 30 years (range 19–45). Almost all (91%) of patients in group A  and in group B were females. The average period of follow-up for the group A patients was 8 months (range 5–17 months), and for group B, it was 5 months (3–11 months). The demographic data and intraoperative findings have been described in the Table 1. The average duration of the surgery in group A was 42 min (range 29–65 min), and in group B, it was approximately 32 min (range 38–70 min). Chorda tympani nerve was found to be injured only in one patient in group B, and it was completely spared in patients with group A. Patients were first evaluated in the postoperative period 6 hours after the surgery. Otosclerotic foci were detected anterior to the oval window in almost all the cases in both the groups, except one patient in group B where the foot plate was found to be thick and obliterative. Stapedotomy was performed after adequate thinning out the footplate with the micro drill. A total 8 (30.76%) patients in group A and 15 (60%) patients in group B had presented with giddiness in the immediate postoperative period where the difference was found to be significant (*p* = 0.03). Similarly, spontaneous nystagmus was detected in 10 patients (4 in group A and 6 in group B) six hours after surgery (*p* = 0.04). The preoperative ABG in group A was 37.51 ± 7.2 dB, and in patients of group B, it was 36.66 ± 8.6 dB. Postoperative ABG in patients of group A and group B was found to be 13.92 ± 6.8 dB and 15.42 ± 7.5 dB, respectively. Similarly, the postoperative AB-closure (ABC) in group A and group B were 23.10 ± 11.64 dB and 22.20 ± 12.24 dB, respectively (Table 2). There was a significant difference between the preoperative and postoperative ABG in each group (*p* < 0.05). Approximatly 70% patients in group A and 76% cases in group B had ≤ 10 dB ABG at the end of 12 weeks follow-up. Again, 92% of the patients in group A and group B had < 20 dB closure of the ABG in the postoperative period (Table 3). The mean DHI score at the end of one week was 43.92 ± 6.63 in group A and 51.68 ± 11.38 in group B, respectively (*p* = 0.00) (Table 4). Similarly, the mean DHI score in group A was 22.38 ± 9.33, and in group, B was 18.64 ± 6.67 at the end of 4 weeks (*p* = 0.10) (Fig. 6). None of the patients in both the group had sensorineural hearing loss (SNHL) till the last follow-up period. Again, when the mean DHI score was compared between the two groups at the end of 12 weeks, it was found to be insignificant (*p* = 0.30). One patient in the gelfoam group had floating footplate in during surgery, which was managed conservatively. The tympanic membrane was injured in 2 patients in the fat sealing group and 4 patients in the gelfoam group during the elevation of the tympanomeatal flap, and none of the patients had any residual perforation till 12 weeks of follow–up. No other major complications were detected either in the intraoperative/postoperative period.

**Table 1:** Demographic data and patients characteristics of the study population (n = 46)

	Lobular fat	Gelfoam	*p* value
	(n = 26)	(n = 25)	*χ*^2^ test
Number of procedures	26	25	
Age (years)	35 (range 22–48)	30 (range 19–45)	
Female (%)	20 (91%)	21 (91%)	
Ear, right (%)	15 (68%)	19 (71%)	
Canaloplasty	01 (3.84%)	2 (7.69%)	0.35
Chorda tympani nerve injury	01 (3.84%)	00 (0.00%)	0.33
Giddiness (After 6 h)	8 (30.76%)	15 (60%)	0.03
Nystagmus (After 6 h)	4 (15.38%)	6 (24%)	0.40
Giddiness (After 1 week)	2 (7.69%)	3 (12%)	0.19
TM injury	02 (9%)	01 (4%)	0.27
TM perforation	00 (0%)	00 (%)	
Follow-up (months)	08 (range 5–17)	05 (range 3–11)	0.46
Operative time (minutes)	42 (range 29–65)	32 (range 38–70)	0.40
Hospital stay (days)	2.38 (range 2–4)	2.72 (range2-4)	0.06

**Table 2:** Pre and postoperative hearing results for lobular fat and gelfoam groups

Characteristics	Lobular fat (n = 26)	Gelfoam (n = 25)	*p*, 95% CI, SEM
*Sealing of foot plate*			
Preoperative ABG	37.51 ± 7.2 dB	36.66 ± 8.6 dB	*p* = 0.70, (CI− 3.6–5.3), SEM = 2.2
Postoperative ABG	13.92 ± 6.8 dB	15.42 ± 7.5 dB	*p* = 0.56, CI (− 5.5–2.5), SEM = 2.0
ABC	23.10 ± 11.64 dB	22.20 ± 12.24 dB	*p* = 0.78, CI (− 5.8–7.6), SEM = 3.3

*CI*; confidence interval, *dB*; decibel, *ABG*; air bone gap, *ABC*; Air bone closure, *SEM*; standard error mean

**Table 3:** Air–bone gap reported in bins of 10 decibels

ABG (dB HL)	Group A (n = 26) Number of patients (%)	Group B (n = 25) Number of patients (%)	*p* value *χ*^2^ test
0–10 dB	18 (69.23%)	19 (76.00%)	*p* = 0.82
11–20 dB	6 (23.07%)	4 (16.00%)	*p* = 0.60
21–30	02 (7.69%)	02 (8.00%)	*p* = 0.97

*dB HL*; Decibel hearing loss, *ABG*; Air bone gap

**Table 4:** Mean DHI scores in the follow-up periods

Characteristics	Lobular fat (n = 26)	Gelfoam (n = 26)	*p*, 95% CI, SEM
Mean DHI score (1 week)	43.92 ± 6.63	51.68 ± 11.38	*p* = 0.00, CI (− 12.9–2.5), SEM = 2.5
Mean DHI score (1 month)	22.38 ± 9.33	18.64 ± 6.67	*p* = 0.10, CI (− 8.1–8.3), SEM = 2.2
Mean DHI score (3 months)	14.80 ± 5.85	15.54 ± 6.10	*p* = 0.66, CI (− 2.6–4.1), SEM = 1.6

*DHI*; Dizziness Handicap Inventory, *CI*; confidence interval, *SEM*; standard error mean

## Discussion

In spite of the advancement in the endoscopic surgical techniques, the audiological outcomes and the postoperative complications with respect to the common tissue seals are not well documented in the world literature. In the present study, we have compared the effectiveness of the gelfoam and the adipose tissue in improving the audiological parameters and in controlling the postoperative giddiness in primary endoscopic stapes surgery. When the AC and BC thresholds were evaluated for both the groups in preoperative and postoperative periods, it had been found that there was a significant improvement in the AC threshold (*p* = 0.00), with significant closure of the ABG (*p* = 0.00) in both the groups. But on comparing the AC and ABG, we did not find any significant difference between them (*p* > 0.05). Again, 69% of the patients in group A and 76% of patients in group B had < 10 dB of ABG and approximately 80% of the patients in both groups had < 15 dB of ABG at the end of 12 weeks in the postoperative period. Similar results were obtained in a study conducted by Faramarzi et al. [9] showing similar ABG in both the fat and gelfoam group (*p* > 0.05). Gelfoam was considered to be ototoxic due to the presence of the formaldehyde used during the sterilization process [10], which has been significantly minimized after the modification in the sterilization, reducing the harmful effect of the later [11]. As described by Shenoi, 20–60 μg of formaldehyde can be harmful to the organ of Corti, and the chance of sensorineural hearing loss will be more with an increment of the sterilizing agent. Meltzer et al. compared vein plug or fat plug with Gelfoam in stapedectomy. They achieved ABG within 10 dB in 71% of the patients, in the vein or fat plug group and 75% in the Gelfoam group [12]. Successful hearing outcomes have also been reported in the previous studies using the fat and gelfoam as sealing material in stapes surgery [13, 14]. In contrast, in the present study, we did not find any patient in the gelfoam group with sensorineural hearing loss till 3 months of follow-ups in any of the groups. Vertigo in the early postoperative period is the commonest morbidity in the postoperative period attributed to the stapes surgery [15–18]. Although the cause of postoperative giddiness is thought to be multifactorial, the loss of perilymph could be the major contributor attributed to this. Various autologous tissue and synthetic materials have been used for sealing of the stapes window to minimize postoperative vertigo, although gelfoam and adipose tissue being the commonest among them. In the present study, 8 (30.76%) patients in the adipose tissue group and 15 (60%) in the gelfoam group had unsteadiness in the immediate postoperative period (*p* = 0.03), who required vestibular sedative. But spontaneous nystagmus was detected in 4 patients in the adipose group and 6 patients in the gelfoam group (*p* = 0.04). Again when the DHI scores were compared between the two groups at the end of 1 week, it was evident that the patients with the adipose tissue group had a significantly lower giddiness score than that of the gelfoam group (*p* = 00). At 4 weeks and 12 weeks of surgery, on comparison of the DHI scores between the two groups, we did not find any significant difference between them (*p* > 0.05). This short term control in vertigo could have been due to the optimized sealing of the stapes window with the adipose tissue, minimizing loss of perilymph. Giddiness in stapes surgery can be seen early [7] in the postoperative period, or it can be encountered in the delayed postoperative period [19], later can delay the hospital stay. As demonstrated in the present study, when the DHI scores were evaluated between the two groups, most of the patients in both the groups became free of vertigo after one week of surgery, but the patients with the fat sealing group had a better vertigo control rate compared to the gelfoam. With respect to the hospital stay, we did not find any significant difference between the two groups, although the mean operative period was longer in the adipose group. Again none of the patients in any the group had sensorineural hearing loss (SNHL) till the 12 weeks of the follow-up period in contrast to the published literature [20]. The tympanic membrane was injured in 2 patients in the fat sealing group and 4 patients in the gelfoam group during the elevation of the tympanomeatal flap, and none of the patients had any residual perforation till 12 weeks of follow-up. According to McNeil, untraumatized adipose tissue and fat can provide an adequate inner barrier for the protection of the inner ear [6]. However, others have claimed that gelfoam produces a thinner membrane than the tissue graft [21, 22]. Although the incidence of perilymph fistula is found to be 0.9% in stapedectomies, it was negligible in stapedotomies surgeries [23] as obtained in the present study. Adipose tissue can be considered a better autograft compared to the gelfoam for sealing of the stapes widow in primary endoscopic stapedotomy, ensuring adequate control of vertigo in the early postoperative period besides significant improvement in the hearing outcome. A large sample size with a long term follow-up may be required for a better understanding of the results.

## Conclusion

Adipose tissue and gelfoam are the commonly used sealing materials in primary endoscopic stapes surgery. Although they have a comparable hearing outcome, the vertigo control in the early postoperative period was significantly better in patients with adipose tissue seal. A large sample size with a long term follow-up may be required for a better understanding of the results.
